# The Significance of Measuring Vitamin D Serum Levels in Women with Uterine Fibroids

**DOI:** 10.1007/s43032-020-00363-8

**Published:** 2020-10-27

**Authors:** Michał Ciebiera, Mohamed Ali, Lillian Prince, Stanisław Zgliczyński, Grzegorz Jakiel, Ayman Al-Hendy

**Affiliations:** 1 Second Department of Obstetrics and Gynecology, Center of Postgraduate Medical Education, ul. Cegłowska 80, 01-809 Warsaw, Poland; 2 Department of Surgery, University of Illinois at Chicago, Chicago, IL, USA; 3 Clinical Pharmacy Department, Faculty of Pharmacy, Ain Shams University, Cairo, Egypt; 4 Biological Sciences Division: Public Health Sciences, University of Chicago, Chicago, IL, USA; 5 Department of Internal Diseases and Endocrinology, Central Teaching Clinical Hospital, Medical University of Warsaw, Warsaw, Poland; 6 First Department of Obstetrics and Gynecology, Center of Postgraduate Medical Education, Warsaw, Poland; 7 Department of Obstetrics and Gynecology, University of Chicago, Chicago, IL, USA

**Keywords:** Uterine fibroid, Leiomyoma, Vitamin D, 25-hydroxyvitamin D, Serum level, Risk factor

## Abstract

Uterine fibroids (UFs) are benign tumors originating from smooth muscle cells and are considered a common pathology that affects numerous women which is a notable socio-economic problem. Several UF risk factors have been identified including black race, obesity, and vitamin D deficiency. Vitamin D is steroid compound with pleiotropic effects on the human body. Vitamin D deficiency is a major public health concern worldwide. Several studies have shown that the majority of UF patients experienced hypovitaminosis D. In addition, sufficient vitamin D serum levels are associated with the reduced risk of UFs. In this review, we present available data highlighting the importance of measuring vitamin D serum levels in women with UFs and women at a high risk for UF development. We proposed a preliminary clinical instruction of 25-hydroxyvitamin D measurements and vitamin D supplementation for clinicians who are involved in the treatment of patients with UFs. Achieving sufficient serum levels of vitamin D might be of interest in patients with UFs. Screening, supplementation, treatment guidelines, and public health strategies for vitamin D deficiency in women with UFs as well as women at a high risk of UF development might be of potential importance as well.

## Introduction

### Uterine Fibroids—Disease Significance

Uterine fibroids (UFs), also known as leiomyomas, are benign tumors originating from smooth muscle cells. They are considered a common pathology that affects many women which is a notable socio-economic problem [1, 2]. Various studies showed that by the age of 50, the prevalence of UFs is around 70% and extends to 80% in African American (AA) population [1, 3]. Several UF risk factors were identified including, most importantly, black race, older age, vitamin D deficiency, obesity, family history, low parity, long period since last labor, food additives or soybean milk consumption, and hypertension [4, 5].

Although commonly benign, UFs are associated with significant morbidity. Although some UF patients may be asymptomatic, 25–50% of them may present a wide range of severe and chronic symptoms, such as abnormal uterine bleeding (AUB), anemia, pelvic pain and pressure, gastrointestinal problems, subfertility, and various obstetric complications [1, 6, 7]. Thus, symptomatic UFs generate an enormous healthcare burden worldwide [1, 8, 9]. Nowadays, the main therapeutic modalities in UF treatment include expensive surgical procedures (hysterectomies or myomectomies). Therefore, there is an urgent need for alternative and preventive therapeutics especially for women who still pursue future fertility plans [8]. According to Merrill et al., UFs are responsible for 30% of all hysterectomies in women of reproductive age [10]. Moreover, US hospital admissions have increased by more than a fifth for 10 years [11].

### Vitamin D Metabolism

Vitamin D is a steroid compound with pleiotropic effects on the human body. Vitamin D receptors (VDRs) are found in various organs including those in the female reproductive tract [12, 13]. Vitamin D exists in two different isoforms: ergocalciferol (vitamin D_2_), which is found in plants or yeast, and cholecalciferol (vitamin D_3_), which is introduced to the body with nutrition or synthesized via ultraviolet radiation [14, 15]. The major sources of vitamin D are fatty fish, cod liver oil, or egg yolks [16]. In some countries, it is also added to fortified milk as well as to some yogurts, juices, and breakfast cereals [17].

Vitamin D deficiency is a major public health concern worldwide [17, 18]. Generally, vitamin D deficiency results from (1) low vitamin D consumption in the diet and low sun exposure, (2) the inability to absorb intestinal vitamin D, (3) the lack of biological vitamin D activation in the kidneys, the liver, or both [19, 20]. Several consequences are associated with vitamin D deficiency including infections, autoimmune diseases, cardiovascular diseases, different types of diabetes, neurocognitive or psychiatric diseases, cancer, or adverse pregnancy outcomes [21-23]. Additionally, recent metaanalyses showed that low serum vitamin D level increased the risk of all-cause mortality [24], while vitamin D supplementation might reduce the risk of death in oncological patients [25].

**Fig. 1:**
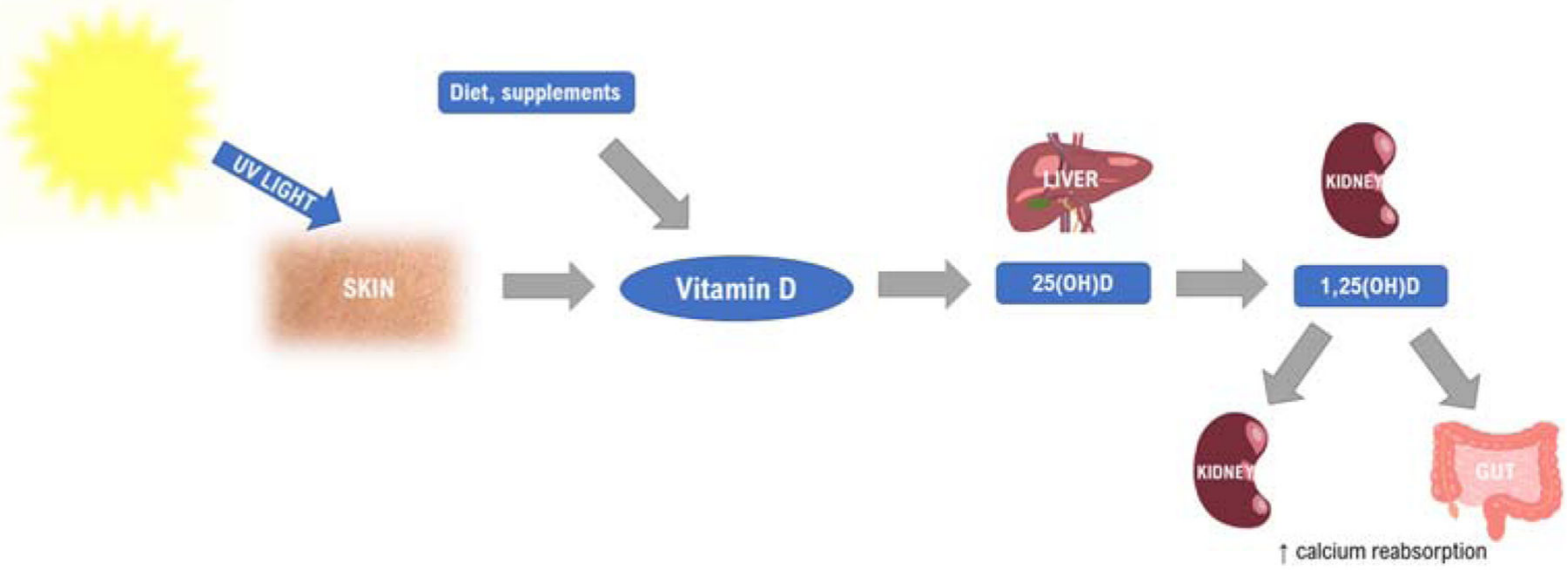
Vitamin D metabolism. UV, ultraviolet; 25(OH)D, 25-hydroxyvitamin D; 1,25(OH)D, 1,25-dihydroxyvitamin D

The metabolism of vitamin D starts in the skin under the influence of sunlight (Fig. 1).

The amount of needed sunlight may vary depending on the exposure time, age, race, clothing, and accompanying medical problems [26]. Nutrition intake traditionally plays a relatively minor role. However, in some areas, due to the lack of sunlight, vitamin D supplementation may be important [17]. Endogenous vitamin D production is limited by factors like geographic latitude, season, weather conditions, clothing, and the use of sunscreens [27]. The biological production of vitamin D changes during the lifespan with the production decreasing with age [14, 22, 26]. People with darker skin pigmentation (e.g., AA or Latinos) need more sun exposure to produce adequate amounts of vitamin D [19].

### Serum Vitamin D Level Clinical Definitions

There is an ongoing debate among experts regarding the identification and terminology of vitamin D deficiency [28-31]. The most accurate way to measure vitamin D serum levels in the human is the 25-hydroxyvitamin D [25(OH)D] blood test [32, 33]. According to the Endocrine Society 2011 guidelines, vitamin D *deficiency* is defined as 25(OH)D levels of 20 ng/mL or lower, *insufficiency* as 21–29 ng/mL, and *sufficiency* as 30 ng/mL or higher [31, 34]. Płudowski et al. described the optimal concentration of 25(OH)D to be ranging from 40 to 60 ng/mL to achieve its best pleiotropic effect while maintaining a low risk of toxicity [28]. The scientific debate among scientific committees responsible for clinical instructions is over the serum level at which vitamin D supplementation is recommended [33]. For example, according to the US Institute of Medicine (IOM), no additional benefit was associated with achieving 30 ng/mL serum 25(OH)D concentrations compared to 20 ng/mL, and this level should be treated as sufficient [30].

### Uterine Fibroids Biology—Overview

UFs are composed of abnormal smooth muscle cells placed in an extensive amount of altered extracellular matrix (ECM) [35-37]. UF growth is hormone-dependent, so UFs are rarely observed in girls before menarche; they become more prevalent among women aged 35–45 and are mostly repressed in postmenopausal women [6, 35]. Estrogen and its receptors were traditionally thought to play a major role in UF growth. Interestingly, new studies showed that progesterone played a significant role in UF growth and estrogen mainly induced progesterone receptor expression [38, 39]. These steroid hormones induce growth factors and cytokines which affect tumor biology and growth as well as the accumulation of ECM [37, 40, 41]. Numerous UF-derived symptoms may be explained by cytokine influence [41, 42]. Transforming growth factor β (TGF-β) appears to be one of the most involved growth factors in UFs considering its role in fibrosis [41, 43]. Additionally, the inflammatory process highly contributes to tumor biology via several cytokines [41, 44].

### The Role of Vitamin D in Uterine Fibroid Biology—Overview

In 2009, active vitamin D was found to effectively inhibit cell growth in vitro [12]. Since then, accumulating data have emerged showing that vitamin D deficiency might be strongly associated with the development and growth of UFs [13, 45]. In 2016, Al-Hendy et al. found a strong connection between vitamin D deficiency and known pathways involved in UF pathology [46]. They showed that UFs with *MED12* gene somatic mutations exhibited the upregulation of the Wnt/β-catenin pathway in comparison with the adjacent myometrium. Moreover, vitamin D treatment inhibited such activation of Wnt/β-catenin and downregulated the expression of mammalian target of rapamycin (mTOR) signaling in both cell types. Those interesting findings suggested that vitamin D might have the potential to inhibit major pathways in UF biology, including those connected with TGF-β [46]. Additionally, vitamin D was reported to have immunologic properties via the vitamin D receptor (VDR) in reproductive tissues [13, 45] as well as anti-inflammatory functions [44, 47]. Recently, the same group has found that vitamin D deficiency might be associated with an increased expression of steroid receptors in murine myometrium in addition to an increased expression of genes related to proliferation, fibrosis, exaggerated inflammation, and DNA damage in murine myometrium [47].

The role of progesterone was mentioned before. Moreover, some findings concerned a potential connection between progesterone and vitamin D pathways that suggested that both steroid hormones cooperated with each other for a more effective regulation of the immune system. For example, it is believed that progesterone induces VDRs in T cells for enhanced regulation by vitamin D and vitamin D is an important regulator of T cell–dependent inflammatory responses [48]. However, data are still scarce in this matter in UF research.

Several studies showed that the majority of UF patients experienced hypovitaminosis D [49-51]. In addition, some authors believe that sufficient vitamin D serum levels might be associated with a reduced risk of UFs [49]. Studies showed vitamin D as a potential single [52] or combination treatment [53, 54]. These topics will be described later on in this manuscript.

A review of the literature indicated that not many studies investigated vitamin D serum levels in patients with UFs. Therefore, it is necessary to conduct additional research to develop recommendations concerning vitamin D screening in women with UFs or at a high risk of developing UFs, similar to recommendations for testing pregnant women who are at an increased risk of vitamin D deficiency (e.g., women with limited sun exposure, women with darker skin). Clearly, several clinicians might be unaware that vitamin D deficiency may contribute to UF development.

### Aim of the Review

In this review, we present the available data highlighting the importance of measuring vitamin D serum levels in women with UFs and women who are at a high risk of their development. We also provide preliminary clinical guidance proposal in this aspect.

## Materials and Methods

This article presents a narrative review on the significance of vitamin D measurements in women with UFs or in women who are at a high risk of UF occurrence. The authors conducted an extensive search in PubMed of the National Library of Medicine and Google Scholar. A literature search was mainly performed using the following keywords: “uterine fibroid” and “vitamin D.” All relevant studies related to UFs and vitamin D published in English until August 2020 were included in this review. We focused on the correlation between vitamin D deficiency and UFs as well as UF-related clinical symptoms. The authors also proposed a preliminary clinical guidance for clinicians who might be involved in the treatment process of patients with UFs or at a high risk of developing UFs.

## Discussion

### Vitamin D Deficiency—Epidemiology and Current Measurement Indications

Low serum vitamin D is a tangible worldwide problem, especially in women from the Middle East as described by Palacios and Gonzalez. Moreover, data are scarce in several countries, mostly in Africa and South America [55]. Surprisingly, the levels of 25(OH)D reported in several studies revealed that the majority of the populations are vitamin D deficient, especially during winter and spring [14, 55, 56].

Low 25(OH)D serum level is a marker of poor health [57], as vitamin D status might be associated with various diseases [24]. Vitamin D deficiency is also a consistent finding across age, ethnicity, and latitude in obese people [58]. These findings might sound intimidating. Many reports and data without any systematic analysis lured many physicians to routinely test 25(OH)D levels in their healthy patients. In fact, current medicine did not show a practical reason for most people to be tested for vitamin D deficiency. According to recommendations published in 2015 by the United States Preventive Services Task Force (USPSTF), whole population screening for vitamin D deficiency is not recommended for healthy, non-pregnant adults or elderly who are seen at family care settings and do not present any signs or symptoms of vitamin D deficiency or conditions for which vitamin D supplementation is recommended [59]. Conversely, 25(OH)D serum level measurements might be important in people who are at risk of deficiency or have medical conditions that increase that risk [19, 33].

There are ongoing scientific discussions and consensus dilemma to determine normal cut-offs for serum vitamin D, as well as the legitimacy of such tests. For example, in France, the indications for serum vitamin D level measurement were restricted only to rickets, osteomalacia, elderly with a high risk of fractures, kidney transplant recipients, and adults after bariatric surgery. According to Souberbielle et al. (2016), such indications were too strict. A group of French clinicians proposed a different approach where bone fragility, chronic renal failure, malabsorption, and other clinical signs suggesting vitamin D deficiency or vitamin D toxicity should also be taken into consideration for vitamin D measurements [60]. Notably, diseases that affect the intestinal absorption of vitamin D should be considered, such as extensive surgical resections, celiac disease, or Crohn’s disease [61].

Recently, in 2017, Sowah et al. found that shift, indoor, and healthcare workers were among the high-risk groups of vitamin D deficiency. It could be corroborated by missing plenty of sunlight exposure during lifetime due to their duties. Some groups of medical students (72%) and resident doctors (65%) were particularly at risk for the same reason [62].

The increased public awareness of vitamin D deficiency and subsequent complications resulted in higher vitamin D consumption. Nevertheless, it should be used following physician consultation, since vitamin D is still a hormone that might exert a potent influence on various tissues.

### Vitamin D and Uterine Fibroids—Current View

Recent preclinical in vitro and animal studies showed that vitamin D is a potent anti-UF agent [13, 63-66]. According to Halder et al. (2011), 1,25-dihydroxyvitamin D reduced ECM-associated protein expression in immortalized human UF cells [67]. Another study by Sharan et al. showed that vitamin D inhibited the growth and proliferation of UF cells through the downregulation of proliferating cell nuclear antigen (PCNA), cyclin-dependent kinase 1 (CDK1), and B cell lymphoma 2 (Bcl2) and suppressed the expression and activity of catechol-O-methyltransferase (COMT) [68]. In 2012, Halder et al. found that vitamin D treatment significantly decreased the size of UFs in the Eker rat animal model through the suppression of cell proliferation [69]. In 2018, Othman et al. reported that UFs contained significantly lower levels of active vitamin D than the adjacent myometrium. The authors suggested that the overexpression of 24-hydroxylaze enzyme might be a mechanism by which those tumors were under the state of hypovitaminosis D [70]. As mentioned earlier, vitamin D reduced Wnt/β-catenin activation and led to the downregulation of mTOR signaling expression [46]. Moreover, in 2018, Elhusseini et al. found that low serum levels of vitamin D in mice were associated with an increased expression of sex steroid receptors in the myometrium and an increased expression of proliferation, fibrosis, and inflammation-related genes. In this study, the authors also presented that vitamin D deficient diet-enhanced DNA damage in the myometrium which may increase the risk of fibroid development later in life [47]. Vitamin D is involved not only in cell cycle regulation and cell differentiation, but it plays an important role in inflammatory and DNA repair processes [42]. More recently, in 2019, Ali et al. found that 75 DNA repair genes were downregulated after VDR knockdown while the expression of 67 of those genes was restored after treatment with vitamin D. Those findings suggested a novel link between DNA damage and the pleiotropic role of vitamin D. The authors concluded that vitamin D suppressed UF phenotype through orchestrated targeting at different networks in DNA repair [71]. A recent animal study by Corachan et al. in 2020 revealed that short-term treatment with vitamin D did not change UF size. However, long-term use of higher doses induced a significant lesion volume reduction via reduced cell proliferation, reduced TGF-β3 expression, and increased apoptosis [72].

Recently, clinical trials exploring vitamin D effect in women with UFs have started. In 2016, Ciavattini et al. showed that vitamin D could reduce disease progression in small lesions [52]. In 2019, Corachan et al. used samples collected from women undergoing surgery and found that an increased proliferation and abnormal functioning of the Wnt/β-catenin pathway played a crucial role in the biology of UFs, whereas apoptosis appeared not to be contributory. Vitamin D showed an anti-proliferative effect through cell growth arrest and the Wnt/β-catenin pathway inhibition. The study suggested that vitamin D itself might play a smaller role in reducing tumor size and it mostly stabilized its volume and prevented further growth [66]. It might suggest combining vitamin D with other agents that induce excessive apoptosis. Ulipristal acetate (UPA) has a proven role as a compound that might reduce UF volume and clinical symptoms. It is a multifactorial agent that works through reducing cell proliferation rate, inducing apoptosis, and regulating ECM remodeling [73]. Interestingly, in 2019, Ali et al. showed that vitamin D combined with UPA significantly reduced cell proliferation compared to UPA alone. Such co-treatment significantly decreased the protein expression of proliferation markers in comparison with UPA therapy alone, along with a significant increase in apoptosis induction. Such a combination also decreased ECM-derived protein levels and diminished the production of proinflammatory interleukins in UFs in comparison with UPA alone (e.g., interleukins 1α, 1β, 6, 8) [53]. The simultaneous use of UPA and vitamin D in humans was described in two cases. The combination presented good clinical effectiveness as the agents shared synergistic anti-fibroid properties [54]. However, recent reports of potential liver toxicity by UPA raised concerns regarding its use [74, 75].

To conclude, the results proved that vitamin D might offer a potential benefit against UF growth. However, the lack of large population-based clinical trials impedes the validation of the preclinical findings on cells and animals [13, 65].

### Vitamin D and Uterine Fibroids—Perinatal Implications

Numerous UF patients with future fertility plans are seeking help from obstetricians/gynecologists. In this review, we focus on UFs and vitamin D, and not pregnancy. We included references for readers interested in the relationship between vitamin D, reproduction, and pregnancy [16, 76-78].

UFs and vitamin D deficiency in pregnant women are separate problems that may cause serious consequences. The first problem of pregnant women who have UFs is connected with a higher risk of spontaneous miscarriages, fetal malpresentation, preterm birth, rupture of membranes, placental abnormalities, emergency cesarean delivery, and postpartum hemorrhage [79]. The second problem is vitamin D deficiency and its complications. Notably, pregnancy is a state of increased calcium demand and fetal vitamin D status is almost completely dependent on the maternal level of vitamin D. The supplementation of vitamin D during pregnancy is necessary, as food sources were found to be inadequate [80].

Therefore, the American Committee of Obstetricians and Gynecologists advised measuring maternal serum 25(OH)D levels and carefully interpreting them in the context of the clinical circumstances in pregnant women at an increased risk of vitamin D deficiency [81]. Similarly, this guideline may be extrapolated to pregnant women with UFs. A combination of UFs and vitamin D deficiency in pregnant women might result in multiple consequences. For example, some complications caused by UFs might be triggered by coexistent vitamin D deficiency and vice versa. In our opinion, fair evidence is available to support the need to monitor vitamin D levels in pregnant women who are at a higher risk of adverse perinatal outcomes [22, 23]. Considering that UFs are also a potential cause of various perinatal complications, vitamin D screening in pregnant women with UFs might be justified.

### Vitamin D and Uterine Fibroids—Available Clinical Data

Recently, numerous studies have identified low serum concentrations of vitamin D as important players in the etiology of UFs [13, 45]. According to available data, cultural and environmental differences might play a major role in UF development [82] and many of them are connected with vitamin D deficiency [49, 82, 83]. For example, Oskovi Kaplan et al. (2018) found that traditional clothing style, low education level, or being a housewife are high-risk factors for UFs [84]. Similar findings were presented by Haq et al. (2018) in which several lifestyle factors such as diet, lack of exercise, cultural habits, and avoiding sun exposure were associated with vitamin D deficiency in women from the Emirates [85].

Although there are some differences regarding vitamin D supplementation geographically, common habits do exist worldwide as well. For example, growing awareness of vitamin D in the general population and over-the-counter vitamin D, sometimes at very high doses, constitute the risk of uncontrolled use and exogenous hypervitaminosis D, resulting in the high concentrations of serum 25(OH)D or free 1,25-dihydroxyvitamin D [1,25(OH)2D], leading to hypercalciuria and, finally, hypercalcemia considering its fat solubility [86]. It is also important to note that different dosing regimens may have various effects on clinical outcomes. A daily dose leads to stable availability of various vitamin D metabolites, so it could be an important explanation for numerous negative vitamin D intervention trials [87].

Several studies connected low levels of serum vitamin D and UF occurrence. Most of those studies focused on AA women, who have an increased risk of UF development [88-90]. Those populations have a 10 times higher risk of vitamin D deficiency and a 3- to 4-fold higher incidence of UFs compared to Caucasians [91]. Moreover, AA developed UFs earlier and patients presented more severe clinical symptoms [49, 89]. Most of the research highlights the fact that vitamin D deficiency occurs more often among AA women due to higher melanin concentrations which results in decreased serum vitamin D production, as well as lower dairy consumption due to lactose intolerance [51].

Several epidemiological studies emphasized the important role of vitamin D deficiency in the development of UFs [5, 49-51]. We present the available, up-to-date data relating to vitamin D deficiency to women with UFs in Table 1.

The data in Table 1 highlight the clear connections between vitamin D deficiency and UF occurrence. However, more research is required in this field. Only Mitro et al. (2015) found no association between low vitamin D levels and the appearance of UFs within the entire population. Interestingly, in this study, the decreased serum concentration of vitamin D was a risk factor of UFs in white women, but no such correlation was observed in black women [92].

Since vitamin D was proved to stop or slow down the growth of UFs both in vitro and in vivo, as well as in limited clinical trials [52, 66], there would be a point in measuring its level in women who were diagnosed with UFs, especially in those who presented with clinical symptoms. If vitamin D deficiency is diagnosed, proper supplementation should be implemented to slow down UF growth. In our opinion, 25(OH)D serum level measurement in selected women could be the simplest, inexpensive, and effective procedure in UF prevention. The same approach may be extended to women who are at a high risk of UFs [13, 45, 102].

### 25-Hydroxyvitamin D Measurements in Women at a High Risk of Uterine Fibroids—Clinical Guidance

Considering the above, herein we propose recommendations for 25(OH)D measurements in patients who are at risk of developing UFs and in UF patients as well. Our clinical guidance on 25(OH)D serum level measurements is presented in Table 2.

It is only a preliminary proposal and an introduction to the topic, as even with available data, still more research is required to provide well-established evidence-based clinical guidelines [13, 45].

### Vitamin D Supplementation in Selected Groups

Generally, the amount of vitamin D sufficient to restore the normal level of 25(OH)D depends on various criteria including age, weight, skin color, sun exposure, diet, and medical conditions. However, a huge gap still exists between recommended vitamin D dose intake and the poor supply in the general population.

The available forms of supplementation and medications include vitamin D_2_ and vitamin D_3_. Vitamin D_3_ is believed to elevate its serum levels more effectively [19]. According to Cochrane review by Bjelakovic et al. published in 2014, vitamin D_3_ seemed to decrease mortality in elderly people, whereas vitamin D_2_ and others did not have statistically significant beneficial effects on the mortality rate [103]. The dose of vitamin D depends upon the nature and severity of the deficiency. A recommended dose of daily vitamin D intake needed to achieve an optimal skeletal effect is about 400–800 international units (IU) [17]. According to Płudowski et al. (2018), guidelines focused on the pleiotropic effects of vitamin D recommended a target 25(OH)D serum level of more than 30 ng/mL, depending on age, weight, additional diseases, and ethnicity. Thus, the recommended daily vitamin D doses range from 400 to 2000 IU. While the natural sources of vitamin D may slightly raise 25(OH)D concentrations, they are still mostly ineffective to maintain the year-round 25(OH)D concentrations [28].

As stated by Rusinska et al. in 2019, there is a great necessity to implement regular vitamin D supplementation with recommended doses and to develop an effective strategy to alleviate vitamin D deficiency [18]. In patients who suffer from severe deficiency, treatment doses are used. The exact guidelines are not the topic of this paper and the reader is advised to check the current guidelines that might differ according to the country, expert groups or medical societies. However, many clinicians use the standard doses of about 7000 IU daily or 50,000 IU per week [18], as such a treatment is easy to implement with available products. It is also of importance to add 1000 mg of calcium (diet and supplementation) each day during deficiency treatment [104] or even 1200 mg [105] or 1300 mg [18] in postmenopausal women as it reduces bone turnover [105].

As for monitoring, vitamin D treatment in deficient or insufficient women, experts advise measuring 25(OH)D serum level after 3 months (depending on clinical situation). Treatment should be continued until 25(OH)D concentration of about 30–50 ng/mL is reached [18], or 40–60 ng/mL as suggested in guidelines by Płudowski et al. [28]. Once achieved, maintenance dose is recommended [18, 33].

Still, the main problem in our field is the lack of consensus about the threshold and optimal levels which is important to perform good quality clinical trials. The consensus in this area may bring great benefits to women with UFs, and probably levels above 30 ng/mL should be of clinical importance in this problem. In our opinion, patients who are at a high risk of UFs, e.g., obese, elderly, nulliparous, of black race, and those with a positive family history or early menarche, should be screened and offered proper treatment with vitamin D, if necessary [5, 13, 106]. For the suggested guidance in different indications, please refer to Table 2.

As mentioned by Mohamed et al. in 2016, the routine screening of pregnant patients for vitamin D deficiency was conducted by large group of clinicians and studies showed the benefits of vitamin D supplementation in those women [107]. Similarly, we believe it will soon become the daily practice of clinicians who treat women with UFs.

Notably, the response to vitamin D medications might differ among populations and even in the same person [108], as up to 25% of humanity present with slow response after standard supplementation doses. We still do not measure the exact vitamin D response index, but it might be of interest for future studies. For example, similar doses of vitamin D in a high responder group may result in high elevations of 25(OH)D serum levels and the subsequent retention of tumor growth, whereas low responders will only gain a slight elevation of 25(OH)D serum level or even no effect at all [108].

## Conclusions

According to recent data and ongoing research, vitamin D seems to be a promising and cost-effective anti-UF agent. More large well-designed randomized clinical studies are needed to explore the efficiency of vitamin D in women with UFs of all ethnicities, especially women of color that have a higher risk of vitamin D deficiency. Thus, vitamin D could become an option in UF therapy with the additional advantage of its beneficial pleiotropic effect. We hope that ongoing studies will provide a reliable answer. So far, limited trials have been conducted with few participants, less diverse populations, or short-term therapy testing.

Achieving sufficient serum levels of vitamin D might be of interest in patients with UFs. Screening, supplementation, treatment guidelines, and public health strategies for vitamin D deficiency in women with UFs as well as women at a high risk of UF development might be of potential importance as well. In this manuscript, we proposed a preliminary clinical instruction of 25(OH)D measurements for gynecologists and other clinicians who are involved in the treatment of patients with UFs.

## Figures and Tables

**Table 1:** 25-hydroxyvitamin D serum levels and uterine fibroids among different populations

Country	Author	Year	Number of patients	Results	Type of study
USA	Sabry et al. [51]	2013	154 104 cases 50 controls	Low 25(OH)D levels significantly associated with UF occurrence and UF volume. Subjects with greater UF volumes had lower vitamin D_3_ serum concentration. Study revealed a significant inverse correlation between serum 25(OH)D levels and UF volume in black patients.	Cross-sectional study
Italy	Paffoni et al. [50]	2013	384 128 cases 256 controls	The mean serum level of 25(OH)D was significantly lower in women with UFs compared to controls.	Case-control study
USA	Baird et al. [49]	2013	1036 cases 620 black 416 white	10% blacks and 50% whites in the examined group had a sufficient serum concentration of 25(OH)D. Subjects with adequate vitamin D levels had a lower odds of UFs compared to the group with 25(OH)D deficiency. Reported association was similar for both ethnic groups. Sun exposure was also correlated with lower odds of UFs.	National Institute of Environmental Health Sciences Uterine Fibroid Study—random selection from available database
USA	Mitro et al. [92]	2015	3590	Insufficient 25(OH)D serum concentration was associated with UF occurrence in white, but not black subjects.	Cross-sectional study
Republic of Congo	Ingala et al. [93]	2016	432 216 cases 216 controls	25(OH)D deficiency, especially using local criteria was observed in patients with UFs.	Case-control study
Italy	Ciavattini et al. [52]	2016	108 53 cases 55 controls	25(OH)D supplementation re-established normal vitamin D serum concentration in subjects with small UFs. In these cases vitamin D supplementation is believed to reduce the progression of the disease.	Interventional study
Indonesia	Masoem et al. [94]	2017	42 21 cases 21 controls	The mean level of 25(OH)D in the UF-positive group was significantly lower compared to non-UFs. No correlation between the serum concentration of vitamin D and the weight of UF mass was reported.	Cross-sectional study
Poland	Ciebiera et al. [5]	2016	188 105 cases 83 controls	Mean 25(OH)D serum levels in subjects with UFs were significantly lower. Higher TGF-β3 serum concentration, BMI, and family history in the UF group were also found as the risk factors of UFs.	Retrospective cohort study
Turkey	Oskovi Kaplan et al. [84]	2018	124 68 cases 56 controls	No association between vitamin D serum concentration levels and size, volume, location, and number of UFs was found.	Cross-sectional study
India	Singh et al. [95]	2019	144 72 cases 72 controls	The mean serum level of 25(OH)D was significantly lower in subjects with UFs. In 62.5% of cases, the concentration of vitamin D3 was below 10 ng/mL. Occurrence of UFs was correlated with decreased serum 25(OH)D.	Cross-sectional study
India	Kumari et al. [96]	2019	80 40 cases 40 controls	UFs were associated with multipara subjects without a medical history of contraceptive pill administration. Significant decrease in vitamin D and calcium serum levels in cases with significant negative association between vitamin D, and the size of UFs was observed.	Case-control study
Iran	Beygi et al. [97]	2019	106 53 cases 53 controls	An association between high vitamin D serum concentration levels and reduced lesion volume was found. Subjects with vitamin D administration had a reduced tumor volume. The number of lesions was not correlated with 25(OH)D.	Randomized controlled trial
Iran	Hajhashemi et al. [98]	2019	69 35 cases 34 controls	UF size in a group with the administration of 25(OH)D was significantly reduced compared to placebo.	Randomized controlled trial
India	Srivastava et al. [99]	2019	90 45 cases 45 controls	Significantly lower mean concentration of 25(OH)D in UF cases compared to controls. UFs’ size increased with low 25(OH)D concentration. Vitamin D deficiency is associated with UF occurrence.	Cross-sectional study
Iran	Arjeh et al. [100]	2020	60 30 cases 30 controls	No statistically significant decrease in the volume of fibroids was observed in vitamin D treatment group. However, a significant increase was observed in the size of fibroids in the control group.	Randomized controlled trial
China	Li et al. [101]	2020	546	Women with UFs had lower serum 25(OH)D levels versus those without fibroids.	Case-control study

**Table 2:** Clinical situations or risk factors that might qualify for 25-hydroxyvitamin D testing

Clinical situation or risk factor	25-hydroxyvitamin D serum level test
Uterine fibroids: Clinically symptomatic Increase in volume Multiple and large burden Pregnancy	Recommended if any factors present
Women of black race with uterine fibroids	
Asymptomatic or smaller uterine fibroids	Depends on the menopausal status and reproductive plans Clinicians should individualize their management, measurements in the presence of additional risk factors (see below)
Premenopausal status	Depends on additional factors and further reproductive plans Clinicians should individualize their management, measurements in the presence of additional risk factors (see below)
>40 years of age	Additional risk factors (stronger)
Positive family history for uterine fibroids	
>10 years since last birth	
Nulliparous	
Chronic hypertension	
Food additives and soybean milk frequent use	
Low sun exposure	
Obesity	
Low physical activity	Additional risk factors (weaker)
Alcohol use	
Red meat–rich diet	
Early menarche	
Other factors	
Previous vitamin D supplementation/treatment without proper control	Also to exclude the risk of potential toxicity
